# Epidemiological Data Management during an Outbreak of Ebola Virus Disease: Key Issues and Observations from Sierra Leone

**DOI:** 10.3389/fpubh.2016.00163

**Published:** 2016-08-08

**Authors:** Kei Owada, Tim Eckmanns, Kande-Bure O’Bai Kamara, Olushayo Oluseun Olu

**Affiliations:** ^1^School of Medicine, The University of Queensland, South Brisbane, QLD, Australia; ^2^Children’s Health and Environment Program, Center for Children’s Health Research, The University of Queensland, South Brisbane, QLD, Australia; ^3^World Health Organization (WHO) Country Office, Freetown, Sierra Leone; ^4^Department of Infectious Disease Epidemiology, Robert Koch Institute, Berlin, Germany; ^5^World Health Organization (WHO), Geneva, Switzerland; ^6^World Health Organization (WHO) Country Office, Kigali, Rwanda

**Keywords:** Ebola virus disease, infectious disease outbreak, surveillance system, epidemiology, public health practice, Sierra Leone, perspective

## Abstract

Sierra Leone experienced intense transmission of Ebola virus disease (EVD) from May 2014 to November 2015 during which a total of 8,704 confirmed cases and over 3,589 confirmed deaths were reported. Our field observation showed many issues in the EVD data management system, which may have contributed to the magnitude and long duration of the outbreak. In this perspective article, we explain the key issues with EVD data management in the field, and the resulting obstacles in analyzing key epidemiological indicators during the outbreak response work. Our observation showed that, during the latter part of the EVD outbreak, surveillance and data management improved at all levels in the country as compared to the earlier stage. We identified incomplete filling and late arrival of the case investigation forms at data management centers, difficulties in detecting double entries and merging identified double entries in the database, and lack of clear process of how death of confirmed cases in holding, treatment, and community care centers are reported to the data centers as some of challenges to effective data management. Furthermore, there was no consolidated database that captured and linked all data sources in a structured way. We propose development of a new application tool easily adaptable to new occurrences, regular data harmonization meetings between national and district data management teams, and establishment of a data quality audit system to assure good quality data as ways to improve EVD data management during future outbreaks.

## Background

Sierra Leone experienced intense transmission of Ebola virus disease (EVD) from May 2014 to November 2015 when the outbreak was officially declared over. The peak of the outbreak, which in total recorded 8,704 confirmed cases and 3,589 confirmed deaths, occurred around November and December 2014 (1–3). Our observation showed many issues in the EVD data management system, which may have contributed to the magnitude and long duration of the outbreak. At the peak of the outbreak in November 2014, although some information on the spatial distribution of known reported cases was available, reliable epidemiological statistics to determine the actual number of confirmed cases and deaths and to effectively monitor the outbreak could not be obtained. This difficulty related substantially to inadequate management and integration of multiple data sources. Furthermore, there was limited knowledge of the epidemiological situation in some districts due to lack of reliable data (2). In collaboration with partner organizations and agencies, especially the Centers for Disease Control and Prevention (CDC) and World Health Organization (WHO), Ministry of Health and Sanitation (MoHS) staff supported district Ebola response teams with cleaning, analysis, and interpretation of the epidemiological data in the field. In this perspective article, we review the EVD surveillance and data management systems, explain the key issues with data gathering in the field, and the resulting obstacles in analyzing the epidemiological data and indicators during the EVD outbreak response work. We make recommendations for establishment of appropriate data management system during mega outbreaks similar to that seen in West Africa from 2014 to 2015.

## Ebola Virus Disease Data Management and Surveillance System in Sierra Leone

### EVD Data Management System

There were two types of data management system used to provide timely situation reports and describe the trends of key epidemiological indicators – The Epi Info Viral Hemorrhagic Fever (VHF, English version 0.9.4.0) line list database (4) and a Microsoft Excel-based line list database.

### The VHF Line List Database

All information collected using case investigation forms (CIF) were intended to be entered into the Epi Info VHF database at district level. The VHF application is a tool developed by CDC, designed to be integrated with Epi Info 7, and used for case and contact tracing data analysis and reporting during outbreaks of VHF (4). The Epi Info team in CDC headquarters quickly adapted the program to enable multi-user data entry and data merging of the district data bases at the national level. However, handling VHF at the district level confronted the users with several problems such as difficulties in detecting double entries and merging identified double entries. Additionally, the majority of district MoHS data managers were not experienced users of the Epi info and the VHF application tool. Furthermore, CIFs arrived late at District Ebola Response Centers (DERC) and, consequently, the VHF database was never up-to-date even in the late stages of the outbreak.

## Microsoft Excel-Based Line List Database

District MoHS data managers used Excel sheets in addition to the VHF line list to provide timely reports of cases and description of trends. Standard visualization techniques in Excel provided simple graphs summarizing data by time, place, and person. The main source of data to produce these reports were the laboratory sample results, which, in addition to sample specific information, had a selected subset of CIF variables such as age, sex, location (district, chiefdom, village), facility, the date of onset of symptom, the date when specimen was collected and received in the laboratory, and the date when specimen was tested.

### EVD Outbreak Surveillance Data Flow

Cases were identified through active case finding done by surveillance and ambulance teams, contact tracers, and by passive surveillance, which included calls from community, headmen, chiefs, and walk-ins at health care facilities. Epidemiological and clinical information of persons who were identified as suspect, probable, and confirmed [case definitions available in WHO report (3)] were collected by the surveillance teams using the CIF, which include date of onset, signs and symptoms, demographic information, date of admission to health care facilities including holding centers, Community Care Centers (CCCs), or Ebola Treatment Centers (ETCs), date of sample collection, date of sample testing, date of discharge from health care facilities, and status of person either alive or deceased. CIF is the most crucial source of information that should capture accurate epidemiological and clinical information of identified persons. The CIFs were brought back upon completion of field investigation by surveillance officers to DERC where data managers enter the information recorded in them into the Epi Info VHF database. The blood sample of a suspected or probable case is accompanied by a copy of the CIF to the laboratory. DERC also received additional information such as live alerts (source of alert, person being seen, classification of person as suspect, and sample taken), death alerts (swab taken and burial team sent), quarantined households (number and food supply), and EVD confirmed cases (number of confirmed EVD positive based on blood and swab samples in health care facilities), which had to be managed on a daily basis. Most DERCs had established a system of manual dashboards to display this information. The information was then transferred to separate Microsoft Excel sheets or Word documents.

### Key Issues and Challenges in EVD Data Management

Multiple surveillance and data management issues were identified during field work in November and December 2014.

## Data Collection

The data collection was frequently incomplete on the CIF and on the databases (for example, many of the records did not have information on date of onset, sex, age, or residence). This is due to organizational problems including lack of supervision and inadequate training of surveillance officers. At least in the early stage of the outbreak, most of the cases were not identified through active case finding due to incomplete information on contacts. Furthermore, burials could not be accurately quantified in the areas known to continue traditional burials. In addition, it was difficult to confirm EVD-related deaths due to inadequate post mortem mouth swab samples. Thus, the number of confirmed EVD-related deaths may have underrepresented the actual number of EVD-related deaths. During the peak of the outbreak, non-EVD deaths due to diseases such as malaria were expected in Sierra Leone. Before the outbreak, malaria accounted for approximately 38% of hospital admissions and was responsible for about 30% of under-five deaths (4). Furthermore, malaria and EVD share common clinical features (3). Consequently, malaria may have caused delays in recognizing EVD confirmed cases, complicated EVD case management, and the classification of EVD-related deaths, since malaria-related deaths may have been misclassified as EVD deaths (5).

## Data Transmission

In the early stages of the outbreak, there was no clear process of how death of confirmed cases in holding centers, CCCs, and ETCs are reported to the DERC, as there was no structured flow of information. Later daily telephone calls were introduced to the DERC alert desk from each health care facility. There was lack of standardization of reporting deaths from burial teams (number of total deaths), the treatment centers (number of EVD-related deaths), and the laboratory (number of confirmed EVD-related deaths based on swab samples). Ideally, for every death, two copies of the original CIF form and for every suspected living case, three copies of the original CIF form should have been filled by the surveillance team. One completed form should have accompanied the sample to the laboratory, one form should have gone to the DERC, and for suspected cases, one additional form should have stayed with the patient. At least in the first period of the outbreak, this process did not function effectively. A lot of information was lost because it was not transmitted to the DERC and because of problems with compilation of data.

## Data Compilation

There was no consolidated database that captured and linked all data sources in a structured way. Each DERC had adopted different methods of recording information on each case. Lack of consistency in the use of the Microsoft Excel-based line list format caused problems with merging and aggregating cases to report critical information such as a total number of new alerts, a total number of cases reported, a total number of blood test taken, etc., to the national level on a daily and weekly basis. For these reasons, much of the data has never found its way into any structured database and may never be recoverable.

## Data Cleaning and Analysis

Data that are incomplete and inconsistent are not suitable for use in outbreak response planning. Such low data quality issues put limitations on measuring critical indicators, such as the case fatality rate (CFR), incidence rate, the average number of contacts per confirmed case, and accurate interpretation of datasets available during the first months of the outbreak. *Inter alia*, this led to an underestimation of deaths in the VHF database throughout the outbreak. This accounts for the underestimation of deaths in the Ebola situation reports. CFR is calculated as number of confirmed EVD deaths divided by number of all confirmed cases. However, given the underestimation of deaths, the CFR may have also been underestimated. An alternative way to calculate CFR is to use only cases with information on final outcome. “Final outcome deaths” is classified when person died of EVD; however, “final outcome alive” is classified only at the end of hospitalization, thus “death or alive” may be overestimated in the cases without final outcome. The degree of overestimation changed over time as the system of data collection improved. In both ways of calculating CFR, there are high risks of bias.

The back log of suspect or probable cases were not being updated and reported causing delay in reclassification of suspect and probable cases. Furthermore, it was not possible to identify, in the database, how many of those identified as suspect and probable cases became confirmed cases over time.

## Data Dissemination

As a result of key data management challenges mentioned above, WHO, together with the MoHS, use the VHF line list database only in addition to the more timely data reported in situation reports, which was generated based on the laboratory results (3). This dependence on the laboratory data never changed in this outbreak even when the number of cases declined to only a few per day. It was not possible to use VHF as the database for day-to-day information exchange because VHF data had many missing variables and was not being updated on time to provide a daily situation report to the national Ebola response center in Freetown.

## Conclusion

Ebola virus disease data collection, collation, and analysis are critical components of the surveillance and epidemiological investigation pillar of the EVD prevention and control strategy; it is, therefore, important to ensure that it is effectively managed. During the latter part of the EVD outbreak, surveillance and data management improved at the district and national levels in Sierra Leone as compared to the earlier stage. However, a complete cleaning and reconciling of all case data was never finalized because data collected at the earlier stage of the EVD outbreak had many missing variables; in many cases, those missing variables were not recoverable. This explains the large number of unclassified suspected cases, even at the end of the outbreak. Ideally, all suspected cases should have been tested and classified as either confirmed or non-EVD case. Cases were doubled entered (laboratory data and CIF data are entered independently) in some cases rather than having the laboratory data added to the CIF (3). Monitoring and interpreting the severity of the outbreak must take into account those potential factors, and it requires combination of well-coordinated and consolidated multiple data sources including observed number of cases and deaths in the VHF database, the number of daily call alerts on the deaths, the number of reported laboratory swab positive results, active case search (field investigation), and review of existing district situation reports.

## Recommendations to Strengthen EVD Data Management During Major Outbreaks

We propose a number of recommendations, which would improve EVD data management during future outbreaks. First, a new application tool, which is easily adaptable to new occurrences (e.g., integrate new parameters and adjust localization lists), with a multi-enter function with possibility for detection of duplication, merging of double entries, generating a list of missing data, and with easy export possibility should be developed. Such tool should be agreed upon by national MoHS and key partners involved in outbreak management and integrated into the Integrated Disease Surveillance and Response (IDSR) system in the countries to improve the availability and use of accurate and reliable surveillance data for timely response. Furthermore, organizations such as WHO, CDC, and NGOs should agree upon a unified EVD data management plan, which all organizations will support and use during EVD outbreaks. Second, early deployment of experienced data managers to support local MoHS staff and NGO employees in the field during an outbreak would ensure early establishment of strong data management systems. Third, regular data harmonization meetings between district monitoring and evaluation officers, national and external data managers should be conducted to improve data quality and consistency at national and district level. Additionally, surveillance officers should be closely supervised, monitored, and trained in the field by the surveillance coordinators and CIF form checked for completeness. Fourth, a disaster data management team should train local surveillance officers and data clerks on how to collect and enter data. Furthermore, regular field epidemiology and data management trainings should be provided for the MoHS staff members at the national, district, and primary health care levels to strengthen IDSR activities, timely outbreak detection, and effective data management. Fifth, a data quality audit system to assure good quality data should be implemented at every stage of an EVD outbreak.

## Author Contributions

All authors listed (KO, TE, K-BK, and OOO) have made direct contribution to the conception and interpretation of data for the work, drafting and revising the work critically, and approved the final version for publication.

## Conflict of Interest Statement

KO and TE worked as consultant epidemiologists to support World Health Organization Ebola Response team, WHO headquarter in Geneva, Switzerland. KO and TE were deployed to Sierra Leone as field epidemiologists to support WHO response to EVD outbreak in districts in November/December in 2014. K-BK was WHO district coordinator of Ebola Epidemic Response, and OOO was national technical coordinator of WHO’s Ebola Epidemic Response. K-BK and OOO are current WHO staff members. The views expressed in this perspective article are those of the authors alone and do not necessarily represent the views, decisions, or policies of the institutions with which they are affiliated and the position of World Health Organization Ebola Response team. The handling Editor EC declared a past collaboration with authors KO, TE, K-BK, and OOO and the handling editor states that the process nevertheless met the standards of a fair and objective review.

## Abbreviations

CCCs, Community Care Centers; CDC, Centers for Disease Control and Prevention; CFR, case fatality rate; CIF, case investigation forms; DERC, District Ebola Response Centers; ETCs, Ebola Treatment Centers; EVD, Ebola virus disease; IDSR, Integrated Disease Surveillance and Response; MoHS, Ministry of Health and Sanitation; VHF, viral hemorrhagic fever; WHO, World Health Organization.
